# Bio-Hydrogen Production Using Landfill Leachate Considering Different Photo-Fermentation Processes

**DOI:** 10.3389/fbioe.2021.644065

**Published:** 2021-11-17

**Authors:** Hind Barghash, Kenneth E. Okedu, Aisha Al Balushi

**Affiliations:** ^1^ Department of Engineering, German University of Technology, Al Athaibah, Oman; ^2^ Department of Electrical and Communication Engineering, National University of Science and Technology, Muscat, Oman; ^3^ Department of Electrical and Electronic Engineering, Nisantasi University, Istanbul, Turkey

**Keywords:** anaerobic digestion, single photo-fermentation, leachate, bio-hydrogen, renewable energy

## Abstract

Recently, it has become imperative to find new sustainable and renewable sources of energy, in order to avoid dependence on non-renewable traditional energy resources. This would help to overcome the depleting of natural resources for energy production. Hydrogen gas production using biological processes is one of the most attractive solutions in this regard, due to its high energy content and ecofriendly nature. Production of hydrogen using single photo-fermentation process and landfill leachate as substrate was carried out in this paper, by utilizing batch bio-reactor and anaerobic conditions. The pH value and temperature, play an essential role in a bio-hydrogen production process. Thus, in this study, the pH values considered were 6, 6.5, and 7.2, respectively, at a controlled temperature of 37 ± 1°C. This study investigated various schemes that have the possibility of producing hydrogen using; landfill leachate alone, with leachate and addition of inoculum such as sewage sludge, and with substrate such as sucrose and glucose. All experiments were conducted with and without mixing, for effective comparative study. Heat and pH pretreatment were applied in each experiment with the objectives of decreasing the activities of methane-producing bacteria and enhancing the activities of hydrogen-producing bacteria. The hydraulic retention time used in this study was 48 h, in order to obtain optimal performance of the schemes employed. Analysis of liquid leachate was performed for each experiment, and based on the obtained results, the maximum yield of hydrogen produced was 5,754 ml H_2_/L, with a medium pH scale of 6.0, fermentation temperature of 37 ± 1°C and constant mixing speed of 100 rpm.

## Introduction

Lately, there has been a tremendous increase in the consumption of energy, due to population growth, and industrialization. Obviously, the energy demand cannot be met using traditional energy sources due to the depletion of natural resources. Consequently, environmental issues such as climate change and global warming, caused by burning of fossil fuels and the huge production of waste in landfills, are bound to occur. There is growing interest towards more sustainable energy sources by many countries, due to recent technologies and developments in renewable energy. The tremendous amount of wastes generated daily in landfills have huge environmental effects. For instance, the emissions of methane, carbon dioxide and other pollutants, contribute directly to greenhouse gas effects. Besides, huge lands are used up due to dumping of waste, causing huge production of landfill leachate. These wastes take huge space and also require proper management. Moreover, in order to prevent the aquifer from any leakages, require intensive energy to be treated and reused. It was recorded that more than 10% of waste dumped in the landfills is food waste which are rich in organic biodegradable compounds (Baawain et al., 2017). In engineered landfills, organic wastes are allowed to decompose biologically to an inert and stable state, producing landfill leachate. The leachate may contain various microorganisms that have potential for hydrogen production.

In light of the above, there is need of finding a new method to manage waste products in such a cost-effective and eco-friendly way, in order to generate clean green energy to meet demands and for sustainable development. Hydrogen gas is one of the most attractive renewable energy sources, apart from solar and wind, because of its huge potential and high energy content. It is a renewable and clean energy source, with zero emission, causing no harm to the environment (Okedu, 2011; Okedu, 2016). Hydrogen is a sustainable energy source with a high energy yield of 122 kJ/g, which makes it a promising replacement for fossil fuels (Muñoz-Páez et al., 2011). There are few researches focusing on hydrogen as a sustainable resource to overcome increasing energy demand in the literature. Many industrial production processes use hydrogen in metal treatment, oil refining, ammonia production, and other applications in food production. Besides, the demand for hydrogen is anticipated to rise noticeably especially in the transportation sector, where it used as a fuel. About 40 million tons of hydrogen per year would be needed to fuel about 100 million fuel cell-powered cars (Nuri Azbar, 2012). Based on this fact, it is imperative to foster research on hydrogen gas production as primary fuel in aircrafts, in order to meet the demand in the transportation sector.

Hydrogen can be generated from many sources which are not renewable; such as natural gas and coal by gasification or steam reforming, which are known as energy-intensive processes. For sustainable hydrogen production, it is paramount to employ a renewable and environmentally friendly resource. The main resource for hydrogen production that has great attention in recent studies, is biomass resource. Biomass is made up of organic materials which are produced by photosynthesis *via* green plants including algae trees, and crops (McKendry, 2002). Using biomass resource for bio-hydrogen production based on feedstock can provide many benefits such as cost-effective, environmentally friendly and emission of no greenhouse gases such as CO_2._

Generally, bio-hydrogen producing sources are classified into three categories; primary production biomasses (crops), secondary producing sources (agricultural lignocellulosic wastes) and producing sources which are preferred mostly due to non-food sources like algae. The main processes used to produce hydrogen are biological processes which generate “bio-hydrogen” and thermochemical processes. Thermochemical processes comprise liquefaction, pyrolysis and gasification, whilst biological processes focus on direct bio-photolysis, indirect bio-photolysis, photo-fermentation, and dark-fermentation and biological water-gas shift reaction. A combination of dark and photo-fermentation (two-stage process) integration, or bio-catalyzed electrolysis (Shah, 2014), would also lead to bio-hydrogen production. There are other options for bio-hydrogen production considering biomass resources such as; organic food waste, lignocellulosic products (wood and wood waste), aquatic plants like algae and water weeds, industrial or municipal solid wastes, and animal wastes (Das, 2003).

Bio-hydrogen technology has significant economic effects, since biological processes are much less energy-intensive compared with electrolysis and thermo-chemical processes. Depending on the characteristics and composition of the feedstock, these processes are applied. For instance, in feedstock which is rich in carbohydrates, bio-processes like dark fermentation and photo-fermentation are used. However, it is crucial to investigate the specifications of the feedstock which includes, the availability of the source, its carbohydrates content, cost and its fermentability (Argun et al., 2016). In the literature, a lot of work has been done on biomass pretreatment at pilot scale. In Reference (Agrawal et al., 2015), a wheat straw and comparative evaluation of commercial enzyme preparations were investigated at pilot scale for biomass saccharification and fermentation. In this paper, the authors suggested that inhibitors could significantly be detrimental effect on enzymes, therefore, to comprehend the dynamics of the between the enzyme inhibitors, critical moderation would help in improving the hydrolysis of the lignocellulosic biomass (LCB) at low enzyme dosage. Also, the enzyme-lignin binding with surfactants topology was investigated in (Agrawal et al., 2017), for enhanced wheat straw saccharification pretreatment in a pilot scale. The study proposed surfactant supplementation as an effective strategy to achieve higher saccharification yield during saccharification. More so, a pretreatment process and its effect on enzymatic hydrolysis of biomass was reported in (Agrawal et al., 2021), where the enzymatic hydrolysis of different biomass at various temperatures and solid loadings was illustrated in order to show their impacts and implications.

There are limited studies on the use of biomass in Oman and so far, there is no report in the literature on the production of bio-hydrogen in Oman. Though the international renewable energy agency (IRENA) (International Renewable E, 2018) has recognized some huge potentials of bio-hydrogen in Oman. There are ongoing unveiled plans to develop a green hydrogen plant in the port town of Duqm, Oman, with a potential electrolyser capacity of up to 500 MW in the first phase (Djunisic, 2020). Therefore, this work would contribute immensely to the literature in bio-hydrogen area of research in Oman in particular and in the Middle East in general.

In this work, the feedstock used is landfill leachate which can be classified as a third producing bio-hydrogen source, because it is not a food source, and has many characteristics for hydrogen production. Bio-hydrogen production using different fermentative processes is integrated with various pretreatment processes based on the type of feedstock used. There are several pretreatments for each type of feedstock and these can be physical or chemical pretreatments. These pretreatments are important and can enhance the production of bio-hydrogen. Therefore, this study tends to investigate the ability of landfill leachate towards hydrogen production using photo-fermentation process. The salient part of this study is that bio-hydrogen production from landfill leachate, will contribute positively to the goals of Oman towards sustainability and initiate bio-hydrogen production industry. In addition, this work would create opening of new opportunities for further researches towards hydrogen production in Oman, for proper and more cost-effective solutions based on landfill leachate. However, developing new technologies to produce bio-hydrogen at high rates and the storage of the produced bio-hydrogen, might be some of the challenges to overcome with time. In this research the bio-hydrogen production was investigated using solid waste leachate mixed with additional substrate to enhance its production. All experiments were conducted using shaking and non-shaking culture in a batch reactor with different initial pH, heat treatment temperatures and mixing rates.

## Materials and Methods

### Collection of Leachate Samples

Leachate samples were collected from the Multaqa sanitary engineered landfill which is located in Al Amerat, Oman. The leachate at the landfill is collected from different cells varying between old and young cells at the landfill. The leachate generated from the landfill was collected by a pipe and stored in a reservoir. The leachate used in this experiment is fresh and was collected from the cells by using a pump. The temperature at the point of collection was (37–39°C ) at Al -Amerat, Oman. The samples were stored at around 4–5°C for later use. Around 250 ml of the sample is withdrawn from the storage and allowed to reach room temperature which is around 21–22°C before further usage. The stored samples were kept in the refrigerator for no more than 2 weeks to avoid any deterioration of the leachate. The measured parameters were compared to the standards in MD 145/93, which regulates wastewater re-use and discharge in Oman.

### Collection of Sewage Sludge Samples

Sewage sludge samples were collected from the Sewage Treatment Plant (STP), at the German University of Technology in Oman (GUtech). The collected samples were instantly stored in a refrigerator with a specific temperature of 4°C for future usage. This exact degree of sample storage temperature was proven from several studies that it is the best degree of temperature for hydrogen holding bacteria to survive.

### pH

The alkalinity and acidity of the feedstock play a crucial role in bio-hydrogen production. The samples of leachate used in this study have high values of pH at approximately 8.8. The pH was measured for the samples at the laboratory using JENWAY pH meter which is calibrated using four buffer solutions. The value of pH was modified using hydrochloric acid and nitric acid, with molarity of 1 M and 65%, respectively.

### Experimental Model

**FIGURE 1:**
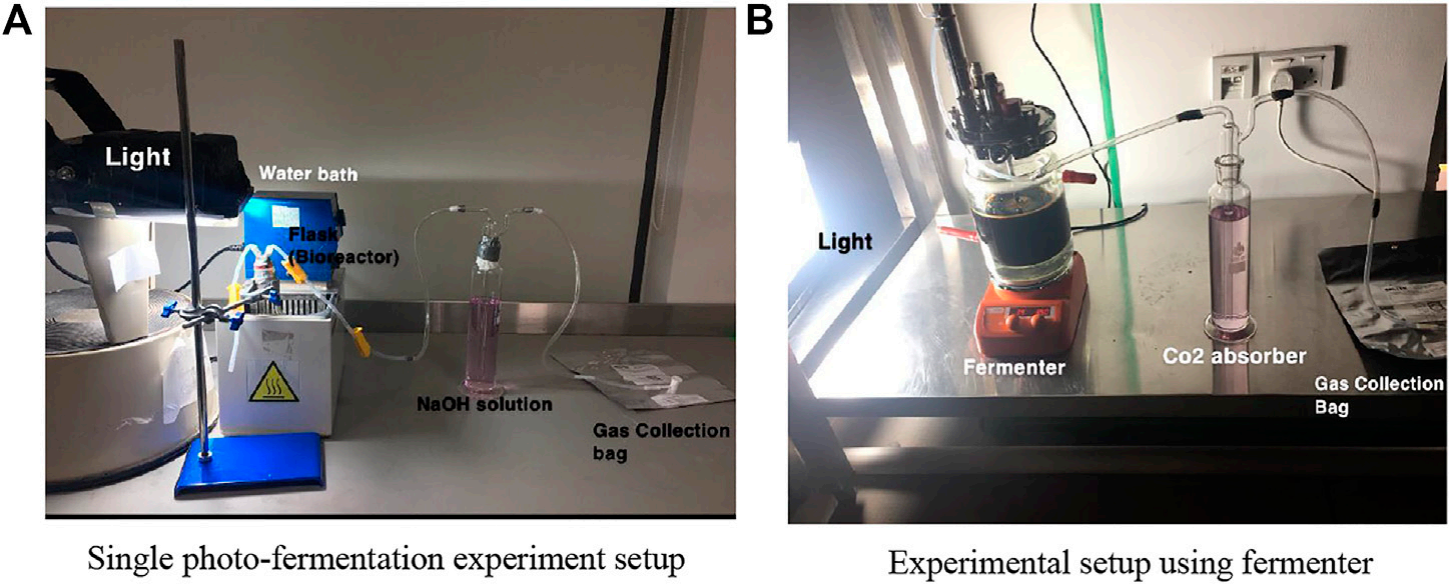
Experimental model system of study.

The experimental setup as shown in Figure 1A for single photo-fermentation process consist essentially of; a round bottom flask with 500 ml capacity which works as a batch reactor, LED light with 150 W, a 300 ml gas washing bottle, 3 L of gas sampling bag from RESTEK 2295, pipes, water bath, heater, magnetic stirrer, nitric acid HNO_3_, 98% sodium carbonate Na_2_CO_3_, plastic clips, lab stand, pH tester, 100% pure nitrogen gas N_2_, and syringe. The batch reactor is placed in a water bath at different temperatures for each experiment. The flask is connected to washing bottle filled with 1 M of NaOH solution and works as CO_2_ absorber. The other pipe which is 4.8 mm thick is connected to the gas sampling bag, where other gases are collected and stored for further analysis.

Figure 1B shows the experimental setup for the fermenter. It consists essentially of a fermenter with; 2,000 ml capacity which works as a batch reactor, LED light with 150 W, a 300 ml gas washing bottle, 3 L of gas sampling bag from RESTEK 2295, pipes, magnetic stirring hot plate, nitric acid HNO_3_, 98% sodium carbonate Na_2_CO_3_, and plastic clips. The fermenter is placed on magnetic stirring plate, where it is adjusted at different rpms. The bioreactor is connected to washing bottle filled with 1 M of NaOH solution and works as CO_2_ absorber. The other pipe which is 4.8 mm thick is connected to the gas sampling bag, where other gases are collected and stored for further analysis. Also, the LED light position is opposite to that of the fermenter and adjusted at illumination of 5,000–6,000 Lux.

Lighting source could have a direct influence on hydrogen producing bacteria. Various studies have been conducted to use distinct lighting sources such as infrared (Argun and Kargi, 2010), halogen (Kim et al., 2012), fluorescent (Argun and Kargi, 2010), and light emitting diode LED (Kawagoshi et al., 2010). However, light-emitting diode (LED) is more stable during operation and has the ability to increase the performance of hydrogen producing bacteria more than the other light sources (Chen et al., 2011).

#### Preparation of Water Bath

In order to make the temperature constant during the photo-fermentation process, the reactor was placed in the water bath. The water bath used is (WCB-6) module from WISE CIRCU. For each litre of distillate water, 1 g of Na_2_CO_3_ was added in order to avoid any deterioration of the equipment. Four litres (4 L) of distilled water was dissolved with 4 g of sodium carbonate in the water bath. The amount of sodium carbonate added was balanced based on the content.

#### Light Intensity Adjustment

Light intensity distribution is a crucial parameter for the experimental setup of the photo-fermentation process, in order to enhance the yield of bio-hydrogen production. Some studies have investigated the effect of using shaking-culture and standing-culture in light illumination.

Based on previous investigation carried out in Reference (Li et al., 2009), the amount of light was adjusted at 4,000–5,000 Lux for non-shaking culture, while for the shaking-culture, the light intensity was adjusted at 5,000–6,000 Lux. The amounts of light received on the surface of the bio-reactor was measured using Robin RT24 light Lux meter environmental monitor.

#### Preparation of Carbon Dioxide Absorber

250 ml of NaOH solution is prepared to absorb CO_2_ which is anticipated to be produced and the remaining gases are collected in the gas sampling bag from RESTEK 22950. To prepare the solution, 10 g of NaOH pellets were mixed with 230 ml of distilled water. Furthermore, 55 drops of Phenolphthalein indicator were added to the solution to indicate the presence of CO_2_.

### Experimental Schemes

Four experimental schemes were investigated in this paper for bio-hydrogen production, for effective comparative study. The four schemes would be discussed in the subsequent section of this paper. The various schemes and their varying conditions are tabulated in Table 1.

**TABLE 1:** Schemes and varying conditions.

Scheme	Working volume (ml)	Hydraulic retention time (h)	Initial pH	Light intensity (lux)		Mixing rate for shaking-culture (rpm)
				Non-shaking culture	Shaking-culture	
1	250	48	6.5	4,000–5,000	5,000–6,000	100, 200, and 400
2	400	48	6.0			
3	900	48	6.0			
4	1000	48	7.2			

All schemes were run for 48 h with and without shaking. For shaking culture, three mixing rates of 100, 200, and 400 rpm were used to conduct all experiments, with the light intensity as given in *Light Intensity Adjustment* of this paper.

#### Production of Hydrogen Using Heat-Treated Leachate Alone (Scheme 1)

The first experiment aims to investigate the possibility of producing bio-hydrogen using leachate alone considering single photo-fermentation process with and without shaking. The working volume was about 250 ml. In this experiment, heat and pH pre-treatments were performed as follows. First and foremost, the leachate was allowed to reach room temperature and then heated at 60°C for 30 min using hotplate stirrers from STUART. Heat pre-treatment is essential to reduce the activity of the bacteria which produces methane and by doing this act, methane will not be produced in the process. After heat treatment, pH of the leachate was measured using JENWAY pH meter and then adjusted at 6.5 using a strong acid 1 M of HCL. The operating temperature of the experiment was 33°C.

#### Production of Hydrogen Using Leachate and Heat-Treated Sewage Sludge and Sucrose (Scheme 2)

In the second experiment, landfill leachate was used as substrate and sewage sludge was added to the batch reactor as inoculum. The sewage sludge was collected and stored as in *Collection of Sewage Sludge Samples*. The sludge was sieved using a 1 mm mesh screen. In order to reduce the activities of the methane-producing bacteria, the sludge was heated at 80°C for 30 min in a water bath. Afterwards, the heat-treated sewage sludge was let to reach room temperature and then mixed with leachate. The leachate was heated at 75°C for 15 min. After the heat treatment was done, the pH was adjusted using HNO_3_ and 1 M of NaOH at 6.0. Then N_2_ (99%) is sparged for 15 min to the 500 ml round flask. The working volume is 400 ml, 20% is sewage sludge and 80% is leachate. Also, 4.5 g of sucrose was added to the batch reactor at the beginning of the experiment. The experiment was run at 37°C. A noticeable production of biogas was shown and collected after 24 h, and bubbles were formed.

#### Production of Hydrogen Using Heat-Treated Leachate and Glucose in Fermenter (Scheme 3)

In the third experiment, a fermenter which has 2 L capacity was used as a bio-reactor. The leachate was heat pre-treated at 65°C for 30 min, Leachate was then mixed with 1.5 M of glucose solution. Afterwards, the pH was measured and adjusted at 6.0, using HNO_3_. Then Nitrogen gas (99%) was sparged for 13 min. The working volume was 900 ml, 80% leachate (medium) and 20% glucose (substrate) and the experiment was studied at 37 ± 1°C.

#### Hydrogen Production Using Leachate and Glucose as Substrates (Scheme 4)

In the fourth experiment, the single photo-fermentation process was conducted using fermenter with 1 L working volume. Leachate was heat-treated at 81°C for 10 min. After the heat treatment, the leachate was allowed to reach room temperature and then a pH treatment was applied. The pH value was adjusted at 7.2, thereafter, 10 g/L of glucose was added. Afterwards, nitrogen (99%) was sparged for 15 min, and the process was conducted at a controlled temperature of 34 ± 1°C.

### Analytical Methods

The collection and preservation measures of the liquid samples were completed according to the Standard Methods for the Examination of Water and Wastewater (APHA) (Eaton et al., 1998). pH level was measured by a 3,510 pH-meter (JENWAY). The produced hydrogen was collected in 3-liter RESTEK 22951 multi-layer foil gas sampling bag. The hydrogen was detected using a gas chromatograph (Agilent, 6890N), which was equipped with thermal conductivity detector (TCD) and a column, using nitrogen as a gas carrier. The flow rate was set as 4 ml/min and the gas is pulled from the bag using a gas syringe of 0.5 ml and then injected in the gas chromatography (GC) at a temperature of 210°C. For measurement, the gas chromatography instrument was calibrated using 20, 40, and 60% of pure hydrogen gas. Moreover, the light intensity of LED light used in the experiment was determined using Lux meter from (Robin RT24 Light Lux meter Environmental Monitor) at the surface of the bio-reactor.

## Results and Discussion

### Characteristics of Landfill Leachate

Table 2 presents the characteristics of the landfill leachate from Alamerat sanitary landfill in Oman for the period of July 2011–February 2019. This is comparable to the standards of discharge of conditions for the treatment, reuse and discharge of wastewater, as per RD 115/2001 (Report, 2018). The decomposition of organic matter generated from the different substances and chemicals, are reflected in the high values of chemical oxygen demand (COD) and biological oxygen demand (BOD), which are 43,128, and 27,181 mg/L respectively. The large numbers of COD and BOD signify that the organic loads disposed in the landfill may be biodegradable (Ahmed and Lan, 2012; Kamaruddin et al., 2017). If the inert/refractory COD is high, this is not biodegradable but may nevertheless be degradable using chemical/heat methods. COD values indicates the contribution or content of biodegradable organic compounds, inorganic oxidizable compounds and non-biodegradable compounds (Christensen et al., 1996; Moreno-Casillas et al., 2007). COD and BOD values are independently related, however, both have an empirical relation which allows BOD value prediction utilizing COD value. The ratio of BOD5 to COD (BOD5/COD) can be used to determine the toxicity and biodegradability of organic loadings which restrict BOD5 testing. Moreover, when the ratio between BOD5/COD is large number, this indicates that the sample contains high biodegradable materials and less toxic. Whereas if the ratio between BOD5 and COD is small number, this indicates that the toxicity of the leachate sample is higher than the organic material (Ahmed and Lan, 2012; Kamaruddin et al., 2017). Leachate is classified as fresh, intermediate or stabilised if its BOD5/COD value is > 0.5, 0.1–0.5 or < 0.1, respectively (Kamaruddin et al., 2017). This method also helps to determine the method of treatment which should be used according to toxicity and organic matter. The ratio of BOD5/COD for Al Amerat landfill leachate is about 0.63, which is a high value and shows that the organic material is highly biodegradable. When compared to the Omani regulations for discharge of non-household liquid waste into sewage per RD 115/2001, the allowable concentration of COD and BOD5 is 200 and 20 mg/L, respectively. The COD and BOD values for the landfill leachate used in this study are higher than the allowable limit. Furthermore, the age of the landfill plays a significant role in the concentration of leachate. Thus old landfills have low value of BOD and COD, whereas for young landfills the values of COD and BOD are higher. For this study, the age of the landfill is not young, it is almost 8 years, but the values of COD and BOD are very high. Therefore, it does not only depend on the age of the landfill. The pH value of the leachate is almost neutral at 7.7, which determines the presence of methanogenic phase. The data also shows the presence of some heavy metals in the samples of leachate. Heavy metals can cause a potential eco-toxicity. Some of these metals are Pb, Cd, Cr, Ni, Cu, and Zn. In this work, the data analysis from Be’ah (Baawain et al., 2017) showed the presence of Pb, Cd, and Cr, with low concentrations at 0.27, 0.02, and 1.31 mg/L, respectively. Also, heavy metals are insoluble and stay at low concentrations during the methanogenic stage (Ahmed and Lan, 2012; Kamaruddin et al., 2017). Moreover, during the acetogenic phase, higher metal solubility in acidic condition contribute to higher concentrations of metals like manganese, iron, calcium, and magnesium in leachate. Al Amerat landfill leachate has high value of ammonia nitrogen, which is about 166 mg/L and this might be due to the composition of waste disposed and the age of the landfill.

**TABLE 2:** Characteristics of raw leachate from Al Amerat engineered sanitary landfill, Oman.

Parameter	Unit	Max	Min	Mean	Standard deviation	Maximum allowable limits A-1	Maximum allowable limits A-2
pH at 25°C	—	9.3	4.7	7.7	1.000	6–9	6–9
COD	mg/L	151,000	2000	43,128	36653.100	150	200
BOD	mg/L	82,500	2	27,181	22444.000	15	20
Suspended Solids (SS)	mg/L	12,350	34	2,134	1975.800	15	30
Conductivity	mS/cm	79	17	44	7.000	0.02	0.27
Total Dissolved Solids	mg/L	100,360	3,910	29,451	9564.900	1500	2000
DO	mg/L	5.7	0.01	0.86	1.300	—	-
Arsenic (As)	mg/L	0.93	0.01	0.22	0.200	0.1	0.1
Boron (Bo)	mg/L	44	1	11.2	4.800	0.5	0.5
Chlorides (Cl)	mg/L	14,442	2,550	8,727	1855.100	650	650
Cadmium (Cd)	mg/L	0.14	0.002	0.02	0.000	0.01	0.01
Chromium (Cr)	mg/L	13.1	0.01	1.31	1.000	0.05	0.05
Iron (Fe)	mg/L	1,055	0.31	183	240.800	1	5
Lead (Pb)	mg/L	2	0.006	0.27	0.400	0.1	0.2
Magnesium (Mg)	mg/L	301	0.04	30	47.000	150	150
Mercury (Hg)	mg/L	4.51	0.001	0.63	1.300	0.001	0.001
Nitrogen (ammonia)	mg/L	800	0.4	166	211.400	5	10
Sodium (Na)	mg/L	22,681	4	5150	2491.000	200	300

### Comparison of the Parameters Used in the Study

The parameters in leachate with the landfill were compared as indicators for acidic and methanogenic phases of the municipal solid waste landfills (Gao et al., 2010; Öman and Junestedt, 2008). The results of Al Amerat landfill show that the samples of leachate are at the end of the acidogenic phase and in the starting methanogenic phase. The value of pH shows the beginning of methanogenic activity. However, for the BOD5 and COD values, the BOD5/COD ratio shows the acidogenic phase, whilst the Fe and Mg concentrations determines a middle methanogenic phase. The variations in the data of leachate such as COD, BOD, heavy metals and pH value are due to the mixing of leachate, which is collected from different landfills with variations in age. According to resources from Be’ah, the leachate reservoir is collected from different cells. Some of them are already considered to be old and closed, while some are new cells. Consequently, it is difficult to indicate, if the landfill is in the acidogenic or methanogenic phase. Table 3 shows the numerical index of comparison for the parameters found in Al Amerat landfill leachate, with landfill phases. Results from this comparative study using different parameters, is found to have variations within the degradation stage of the landfill (Ehrig, 2005; García-Sánchez et al., 2018). All values in Table 3 are in mg/l except pH and BOD/COD.

**TABLE 3:** Comparison of the parameters in leachate with the landfill phase.

Parameter	Acid phase		Methanogenetic phase		Al Amerat landfill leachate	
	Mean	Range	Mean	Range	Mean	Range
PH	6.1	4.5–7.5	8	7.5–9	7.7	4.7–9.3
COD	22000	6,000–60,000	3000	500–4,500	43128	2000–151,000
BOD	13000	4,000–40,000	180	20–550	27181	2–82,500
BOD_5_/COD	0.58	—	0.06	—	0.63	—
Fe	780	20–2,100	15	3–280	183	0.31–1,055
Mg	470	50–1,150	180	40–350	30	0.04–301

### Characteristics of Sewage Sludge as Inoculum

Sewage sludge was obtained from the tank of a municipal anaerobic digester at the German University, Halban, Oman. The sewage sludge had Total Suspended Solid (TSS), Total Dissolved Solids (TDS), and Total Volatile Solids (TVS) of 510, 9,180, and 1,070 mg/L, respectively. Also, the BOD was 248.16 mg/L and COD was 950.53 mg/L.

### Production of Bio-Hydrogen Using Single Photo-Fermentation

Biohydrogen production using photo-fermentation is one of the most popular bioprocesses used to produce hydrogen, where the purple non-sulfur photosynthetic bacteria grow on organic acids like lactic acid, butyric acid, and acetic acid and generate photosynthetic biohydrogen using light under anerobic conditions (Uyar et al., 2012). However, there are various microorganism which are responsible for biohydrogen production using photo fermentation such as: cyanobacteria (blue-green algae), aerobic green algae, anaerobic photosynthetic bacteria, and photosynthetic purple bacteria which are been recognized as the appropriate type for biohydrogen production because they can use byproducts from dark fermentation (as organic acids) or industrial wastes through nitrogenase enzyme (Gil et al., 2013).

There are two enzymes which are responsible for producing hydrogen which are hydrogenase and nitrogenase enzymes, where hydrogenase enzyme is capable for producing and consuming molecular hydrogen which rely on the type of physiological and hydrogenase conditions, while the nitrogenase enzyme have the ability to produce hydrogen in limiting nitrogen conditions working as ATP-dependent hydrogenase (Uyar et al., 2012).

In addition, there are various types of photosynthetic bacteria recorded to be hydrogen producer, but among them the purple non-sulfur bacteria are widely known and well characterized than the others. There are many non-sulfur bacteria which used in many biohydrogen production studies, for example, Rhodospirillum rubrum, Rhodoseudomonas palustris, Rhodobacter sphaeroides, and Rhodobacter capsulatus are the most utilized strains (Dubbs and Robert Tabita, 2004). These bacteria preferred to grow in the existence of small organic acids and in the organic carbon (ANDROGA, 2014). Purple non-sulfur bacteria (PNSB) have the ability to grow under fermentative, photoautotrophic, respiratory, and chemotrophic conditions, which depends also on light availability, type of carbon source and presence of oxygen (Koku et al., 2002).

Photo-fermentation process can be applied to different process conditions such as continuous or batch mode under solar illumination or using artificial light sources and using a wide variety of nitrogen and carbon sources which includes wastewater, food waste and dark fermentation effluents (Uyar et al., 2012). Also, there are various parameters which can influence the activity of biohydrogen producing bacteria and biohydrogen production yield which includes physiological parameters for instance temperature, pH, Composition of the medium, and control of light intensity (Uyar et al., 2012). Moreover, there are several parameters which can also affect the production of biohydrogen in purple non-sulfur bacteria which are insufficient hydrogen generation capacity of nitrogenase, hydrogen is consumed by uptake hydrogenase enzyme, nitrogenase towards ammonia sensitivity and restricted flow of electrons to nitrogenase (Kars and Gündüz, 2010).

Producing biohydrogen using photo-fermentation process and purple non-sulfur bacteria has many benefits such as, higher production of biohydrogen yields than using dark fermentation, usage of purple non-sulfur bacteria in treating wastewater and using various wavelengths of light (Levin, 2004; Tao, 2008; Debabrata and Kaushik, 2004).

#### COD Removal in the Fermentation Schemes

**FIGURE 2:**
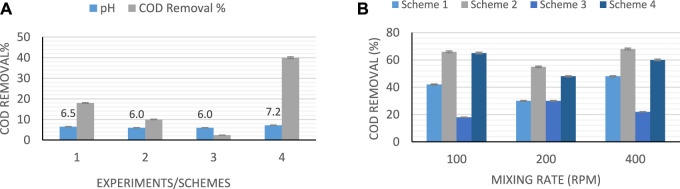
COD removal (%) after each experiment: **(A)** standing-culture **(B)** shaking**-**culture.

Any changes in the value of COD of the feed can have an immediate effect on the activities of the microbial community and the generation of the hydrogen (Gao et al., 2010). Hence, in this study, COD was measured before and after each experiment. The reduction in value of COD illustrates the consumption of the substrate. However, if the percentage of COD removal is low, it indicates the complexity of organic material in the leachate. Figure 2 shows the COD removal (%) after each experiment for standing-culture and shaking-culture, respectively. For this study, it was observed that at high values of pH, the removal efficiency of COD is higher than those with low pH in both shaking and standing-culture. The maximum COD removal percentage was found to be 68% at the highest pH, and the highest intensity used, with shaking at 400 rpm as shown in Figure 2. Previous studies on bio-hydrogen production using photo-fermentation have shown COD removal efficiency of 77%, when the light intensity was higher, which is due to the use of short-chain organic acid (García-Sánchez et al., 2018). Hence, light intensity has a direct effect on the COD removal.

#### pH Values in the Fermentation Schemes

It was observed that there are changes in the value of pH after each experiment. For example, in the first experiment (scheme 1), the pH increased by 19%, while in the second experiment (scheme 2), it was elevated by 40.7%. However, in the fourth trial (scheme 4), the mixture of leachate and glucose was adjusted at 7.2, and after the process completed in 48 h, the pH increased by 15.2%. Usually, in complex mediums like leachate, which is formed by the decomposition of proteins, the bacteria uses the nutrients that exist in the medium and they split off the ammonia from amino acids. Then ammonia is produced and attracts the proton to form ammonium in the solution, which causes an increase in pH. Also, the degradation of carbon source in the medium as the process proceeds, causes an increase in the alkalinity of the medium. However, high final values of pH indicates high increase in alkalinity and reduction in hydrogen production. This is because lower values of pH triggered the generation of hydrogen (Masset et al., 2012).

#### Evaluation of Hydrogen Gas Production Considering Different Fermentation Schemes

**FIGURE 3:**
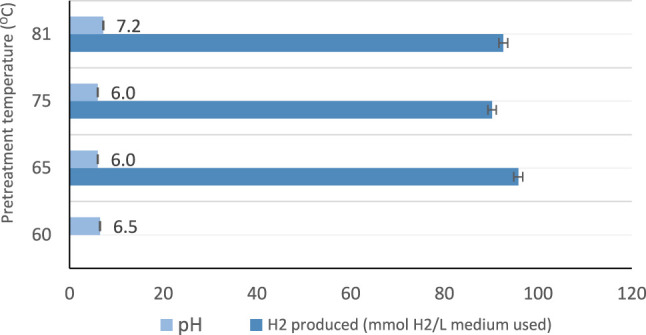
Effect of heat pretreatment and pH on hydrogen produced (without mixing).

The summary of the maximum hydrogen production from each experiment (scheme) is shown in Table 4. Figure 3 shows the effect of heat pretreatment and pH on hydrogen produced without mixing. It was observed that the initial value of pH has a significant influence on the amount of hydrogen gas produced.

**TABLE 4:** The impact of pH values in biohydrogen production.

Inoculum	Substrate	Reactor type	pH optimal	Optimal hydrogen production	References
Mixed cultures	Sucrose	Continuous	4.2	1.61 mol H_2_ /mol glucose	Mu et al. (2006)
Landfill leachate sludge	Glucose	Batch	6.0	6.29 mol H_2_ /mol glucose	Wong et al. (2012)
Anaerobic sludge	Sucrose	Batch	5.5	3.7 mol H_2_/mol sucrose	Wang et al. (2005)
Anaerobic sludge	Glucose	Continuous	5.5	2.1 mol H_2_/mol glucose	Fang and Liu, (2002)
Mixed cultures	Sucrose	Continuous	7.0	1.61 mol H_2_ /mol glucose	Mu et al. (2006)
Anaerobic digester sludge	Rise Slurry	Batch	4.5	346 ml H_2_/g starch	Fang et al. (2005)
Enterobacter cloacae IIT-BT 08	Sucrose	Batch	6.0	29.63 mmol H_2_ /g dray cell-h	Kumar, (2000)

Monitoring the value of pH during the biohydrogen production using photo fermentation is an essential step. pH value can affect the activity of hydrogenase enzymes and influence metabolic pathways (Wang and Wan, 2008). Many studies have investigated biohydrogen production from solid wastes and concluded that controlling pH was crucial to hydrogen production (Dawei et al., 2008). Also, some studies demonstrated that increasing the value of pH may enhance the activity of biohydrogen-producing bacteria to produce biohydrogen during fermentative hydrogen production. However, very high values of pH could reduce the ability of biohydrogen producing bacteria and consequently decreasing the production of biohydrogen (Wang and Wan, 2008). Also, pH influence the synthesis of protein, efficiency of substrate/feedstock metabolism, storage of energy and the microbial metabolites (Yaswanth, 2018). pH values of 4.5–6.5 are considered to be ideal for biohydrogen production, which prevent the activity of methanogenic bacteria which is responsible for growing and enhancing microbial metabolites (Yaswanth, 2018). pH values at this range, enhances and makes active the enzymes for biohydrogen at these values, while at values below four this enzyme “hydrogenase” is influenced. Thus, pH values in the range of (4.5–6.5) is the appropriate condition for biohydrogen production because the biohydrogen generating acidogenic bacteria is found to perform well at this range (Yaswanth, 2018). In contrast, at values higher than 7, methanogen activity is very high where methane is produced. There are many studies that investigated the biohydrogen production using different reactors types such as continuous and batch considering the pH values. Table 4 below is demonstrating some results of studies using different substrates, process condition and the impact of pH values in biohydrogen production.

**FIGURE 4:**
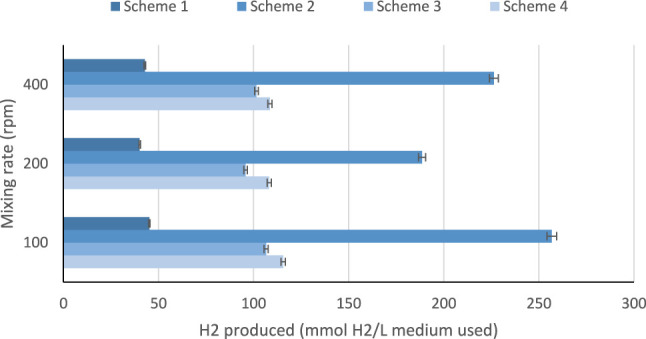
Effect of heat pretreatment, pH, and mixing rate on hydrogen produced.

High initial pH values of the medium showed high hydrogen production at 48 h hydraulic retention time as illustrated in Figure 3. It was observed that pH values of 4.5–6.5 are considered to be ideal for bio-hydrogen production because the bio-hydrogen generating acidogenic bacteria is found to perform well at this range (Yaswanth, 2018). From Table 5, the maximum hydrogen produced was about 5,754 ml H_2_/L medium in scheme 2, which was achieved using batch reactor and leachate. Scheme 2 uses a sewage sludge and sucrose as substrates, considering continues mixing of the medium at 100 rpm as shown in Figure 4. Using heat-treated leachate as substrate and glucose with continuous mixing of the medium at 100 rpm, gave 2,589.2 ml H_2_/L for scheme 4, in Table 5 with better hydrogen gas production than scheme 2 as illustrated in Figure 4. From Figure 4, although the mixture with 400 rpm gave high performance of hydrogen production yield, however, it is not cost effective because of the high mixture rate involved compared to 100 rpm.

**TABLE 5:** Summary of the maximum hydrogen gas produced at each experiment (scheme).

Scheme	Amount of medium used in each scheme (ml)	Gas produced (ml H_2_/L of medium used)	Hydrogen in mmol H_2_/L medium used	Hydraulic retention time (h)	Initial pH	Mean/standard deviation	Light intensity (lux)	Reactor type
1	250	1011	45.1	48	6.5	6.425/0.0375	5,000–6,000	Batch with stirrer (100 rpm)
2	400	5754	256.8	48	6.0	6.425/0.2125	5,000–6,000	Batch with stirrer (100 rpm)
3	900	2390	106.6	48	6.0	6.425/0.2125	5,000–6,000	Batch with stirrer (100 rpm)
4	1000	2589.2	115.5	48	7.2	6.425/0.3875	5,000–6,000	Batch with stirrer (100 rpm)

The photo fermentation process required a source of light either using natural light from the Sun or artificial light using lamps such as fluorescent or tungsten light. The type of artificial light supply, illumination intensity and the distance from the light source to the bioreactor are all influencing the hydrogen producing bacteria. Light intensity distribution is a crucial parameter for the setup of photo fermentation process, to enhance and increase the yield of biohydrogen yield production. Also the type of culture used affects the production rate. Some studies have investigated the effect of using shaking-culture and standing-culture with changing the light illumination, which concludes that the hydrogen producing bacteria was active and optimal hydrogen production found when the illumination varied between 6,000–8,000 Lux with shaking-culture, but for non-shaking culture a higher production yield was found at 4,000–5,000 Lux (Li et al., 2009). Moreover, some researchers have investigated the effect of changing light intensity of incandescent lamps (60 W), from 2000 to 8,000 Lux and the distance from the bioreactor using mixed dark fermentative bacteria and photo-fermentative bacteria and, the highest biohydrogen yield recorded when the light intensity was 8,000 Lux, but while increasing the light intensity further to 10,000 Lux, a notable reduction in biohydrogen production was recorded and this was due to the shade caused by dark fermentative bacteria (Ding et al., 2009a). Thus, biohydrogen generation using photo-fermentation is limited by the availability of light intensity and ensuring continuous lighting is essential for obtaining higher values of biohydrogen yield (Argun and Kargi, 2010).

The light conversion factor affects the microorganisms in photo-fermentation processes. When the light intensity was adjusted at 5,000–6,000 Lux, higher hydrogen production yield was obtained. Furthermore, while using a batch reactor with continuous stirring, high production was achieved at light intensity of 5,000–6,000 Lux and 100 rpm in all the experiments conducted. No hydrogen production was observed at 4,000–5,000 Lux, with the use of standing-culture landfill leachate alone. Similar investigations carried out in the literature concluded that the hydrogen producing bacteria was active and optimal hydrogen production was achieved when the illumination is varied between 6,000–8,000 Lux for shaking-culture. For non-shaking culture, high hydrogen production yield was found within the range of 4,000–5,000 Lux (Li et al., 2009). The results obtain in this study is in line with those of the literature, where the application of 100 rpm for the culture is recommended and showed higher hydrogen production (Reungsang et al., 2007; Ding et al., 2009b). The heat pretreatment demonstrated a noticeable change in hydrogen production. One of the main physical pretreatment which involves thermal changes is heat pretreatment, which can be done by preparing the feedstock used at certain temperature for a certain time. Heat pretreatment is one of the most popular pretreatments used in many studies due to its effectiveness, simplicity and cost-effectiveness which is used to enhance biohydrogen producing bacteria using mixed cultures (Wang and Wan, 2008). Some studies have investigated biohydrogen production using preheated seed sludge at 65°C which has shown a production yield of about 2.30 mol H_2_/mol. Preheating of seed sludge at this temperature have eliminated hydrogen consuming bacteria while enhancing and preserving biohydrogen producing bacteria. Also, it been demonstrated that, preheated seed sludge has highly increase the removal of COD in wastewater (Wong et al., 2014). Reducing COD in wastewater during the production of biohydrogen indicates the potential of using heat pretreatment for treating wastewater using dark fermentation process (Wong et al., 2014). In addition, heat pretreatment plays a significant role in changing the metabolic pathway, when increasing the temperature from 65 to 120°C resulted in pathway change from ethanol-type to acetate for biohydrogen production (Wong et al., 2014). A study which used no heat pretreatment of the mesophilic sludges showed fermentation resulted in the production of carbon dioxide and methane and no hydrogen was produced (MASSANETNICOLAU et al., 2008). Heat pretreatment varies according to the substrate used, for example, if the substrate contain carbohydrates there will be an enhancement in the production of biohydrogen while if the substrate content consists of lignin, cellulose and hemicellulose the mixed culture will consume the produced biohydrogen (Pachapur et al., 2019). Producing biohydrogen at high temperatures for instance thermophilic conditions is significant in increasing the kinetic reactions towards hydrogen production and perfect to inhabit methanogens phase. However, there are some substrates which has lack of simple sugars and complex and diverse microorganisms in the substrates which contribute to change the metabolic pathway of mixed culture to solvent generation and resulted in the reduction of biohydrogen production (Pachapur et al., 2019). Thus, pretreatment of such substrates is essential step in order to enhance and improve hydrogen production. An example of these substrate sources found in literature are solid waste, cow dung, and sewage sludge (Pachapur et al., 2019). A study done on investigation of heat pretreatment on fermentative biohydrogen production, shows that the largest hydrogen yield achieved after heat pretreatment at 65 and 80°C with the hydrogen yield of 6.29 and 6.07 mol H_2_/mol glucose respectively (Wong et al., 2012). While lower hydrogen yield observed at lower temperatures at 40 and 50°C concluded that these temperatures are not effective to enhance the production of biohydrogen and while using seed sludge without heat treatment hydrogen produced was about 2.91 mol H_2_/mol glucose (Wong et al., 2012). Finally, the study on biohydrogen production from landfill leachate have concluded that the biogas collected analysis showed that hydrogen produced from all samples which were preheated at different temperature, some produced higher hydrogen at specific temperatures and some produced lower hydrogen and carbon dioxide and no methane detected which indicates that the process occurred in the absence of methanogenic activity of landfill leachate sludge (Wong et al., 2012). The temperature of heat pretreatment has a dramatic effect to the activity and behavior of microbial community of the substrate used and the energy produced. However, at very high temperatures for example, above 90°C the production of hydrogen from sludge is found to be lower than the hydrogen produced by untreated sludge. In this study, it was observed that the best performance was obtained at heat pretreatment of the leachate at 65–81°C, while the least amount of hydrogen produced was found at heat pretreatment of the leachate at temperatures below 60°C as shown in Figure 3. Thus, from Figure 3, it can be seen that heat treatment of leachate in the range of 65–81°C, is most preferable, with pH range of 6.0–7.2.

**FIGURE 5:**
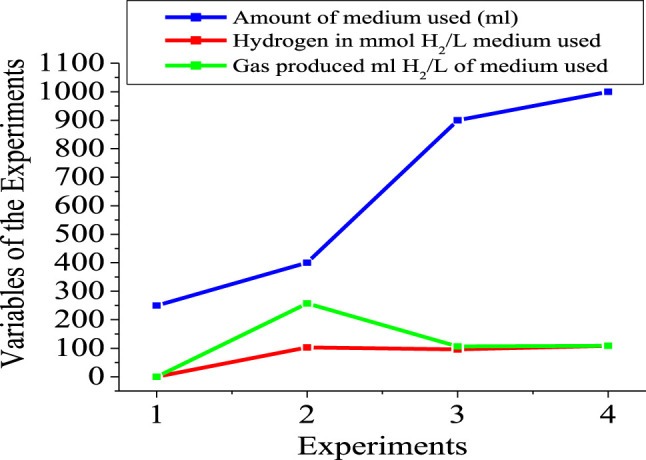
Amount of medium and hydrogen variables of the experiments.

**FIGURE 6:**
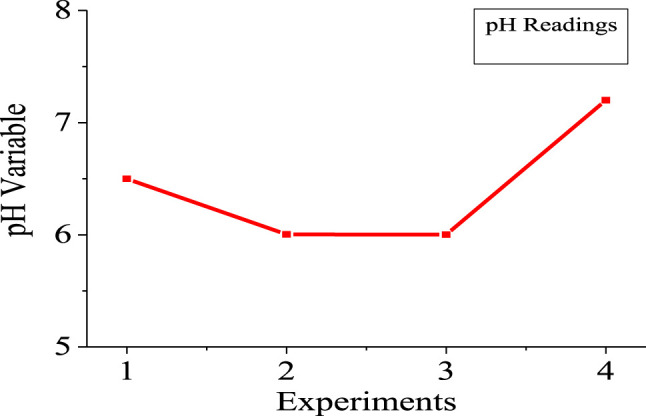
pH variable of the experiments.

Figures 5, 6 show the numerical index and responses of some of the parameters for the experiments. In Figure 5, the amount of medium used exceeds that of the gas produced and the amount of hydrogen used, respectively. Experiment 4 requires more medium amount, compared to experiment 1. However, more gas was produced in experiment 2 compared to the other experiments. Consequently, more hydrogen gas was produced in experiment 2, due to more gas production for experiment 2. Figure 6 shows that experiments 2 and 3 have almost the same pH compared with experiments 1 and 4, respectively. The highest pH value occur in experiment 4, with more medium amount.

The results showed that, bio-hydrogen can be produced using landfill leachate alone, with the addition of inoculums/substrates such as sewage sludge, sucrose, and glucose. Higher production of hydrogen was achieved while mixing leachate with sewage sludge, resulting in the production of 5,754 ml H_2_/L medium, in a batch reactor with continuous mixing at 100 rpm. Higher production was also observed using more leachate with mixing of landfill leachate with glucose, resulting in the production of 2,589.2 ml H_2_/L, in a batch reactor with continuous mixing at 400 rpm.

This study showed that hydrogen production can be achieved with heat treatment at temperature range of 60–81°C with and without mixing. Adjustment of pH at the beginning of the process is an essential step, however, it is recommended not to use pH higher than 7.5, due to low hydrogen production yield. Furthermore, the study showed that hydrogen production using a single photo-fermentation process is limited by the amount of light subjected to the bio-reactor. Therefore, it is important to ensure that the illumination is reaching the culture in the range of 4,000–6,000 Lux. This study concluded that no hydrogen production is observed using leachate alone in standing-culture, below intensity range of 4,000–5,000 Lux.

In this study, a reduction in the COD of the leachate is observed at the end of each process. Maximum COD removal of 66 and 68% was observed using leachate mixed with sewage sludge in a batch reactor with continuous mixing at 100 and 400 rpm, considering light intensity of 5,000–6,000 Lux. Also, because the photo-fermentation process depends heavily on the light intensity, the dark color of the leachate might be the reason for less production, due to low penetration of light to the rest of medium. Higher concentration of ammonia in the leachate might be the reason for the lower production because ammonia can reduce the activity of hydrogen enzymes and inhabit the nitrogen enzymes.

## Conclusion

In this study, the ability of using landfill leachate as feedstock to produce clean and green hydrogen gas considering different photo-fermentation processes were investigated. Bio-hydrogen cannot be produced using landfill leachate alone in standing culture, without the addition of inoculums/substrates or modifying the microbial community. Remarkable hydrogen production was observed in all the experiments/schemes employed in this study, considering continuous mixing at different mixing rates. However, higher hydrogen production is expected with microbial identification for bio-hydrogen gas production. Microbial identification for optimum bio-hydrogen production will be the future scope of this work for optimum culture and process conditions.

## Data Availability

The raw data supporting the conclusion of this article will be made available by the authors, without undue reservation.
